# *Rosmarinus officinalis* L. increases *Caenorhabditis elegans* stress resistance and longevity in a DAF-16, HSF-1 and SKN-1-dependent manner

**DOI:** 10.1590/1414-431X20165235

**Published:** 2016-08-01

**Authors:** D.C. Zamberlan, G.P. Amaral, L.P. Arantes, M.L. Machado, C.R. Mizdal, M.M.A. Campos, F.A.A. Soares

**Affiliations:** 1 Centro de Ciências Naturais e Exatas, Departamento de Bioquímica e Biologia Molecular, Programa de Pós-graduação em Ciências Biológicas: Bioquímica Toxicológica, Universidade Federal de Santa Maria, Santa Maria, RS, Brasil; 2 Departamento de Análises Clínicas Toxicológicas, Centro de Ciências da Saúde, Universidade Federal de Santa Maria, Santa Maria, RS, Brasil

**Keywords:** Caenorhabditis elegans, Natural compounds, Rosemary, daf-2, Stress resistance, Aging

## Abstract

Improving overall health and quality of life, preventing diseases and increasing life expectancy are key concerns in the field of public health. The search for antioxidants that can inhibit oxidative damage in cells has received a lot of attention. *Rosmarinus officinalis* L. represents an exceptionally rich source of bioactive compounds with pharmacological properties. In the present study, we explored the effects of the ethanolic extract of *R. officinalis* (eeRo) on stress resistance and longevity using the non-parasitic nematode *Caenorhabditis elegans* as a model. We report for the first time that eeRo increased resistance against oxidative and thermal stress and extended *C. elegans* longevity in an insulin/IGF signaling pathway-dependent manner. These data emphasize the eeRo beneficial effects on *C. elegans* under stress.

## Introduction

Aging and lifespan of multicellular organisms are affected by several genetic factors. Signal transduction pathways that regulate gene expression in response to extracellular cues are common targets in the search for longevity genes (1). Insulin/IGF signaling is a conserved signal transduction pathway that regulates growth and anabolic functions of multicellular organisms at the expense of cellular stress defenses and repair by modulating stress resistance and longevity (2).

*Rosmarinus officinalis L. (Labiatae)*, popularly known as rosemary, is a common household plant grown in many parts of the world. Aqueous and ethanolic extracts of *R. officinalis* have been shown to contain many substances with pharmacological properties. Health benefits include the following characteristics: antioxidant, antidiabetic (3), hepatoprotective (4), antithrombotic (5), antinociceptive (6), anti-inflammatory (7), antidepressant (8), and gastroprotective (9). Given that *R. officinalis* appears to have beneficial effects in diseases that are strongly linked to aging, we investigated the effects of the ethanolic extract of this plant (eeRo) on aging using the non-parasitic nematode *Caenorhabditis elegans* as a model.

*C. elegans* has been shown to be a valuable model in understanding the molecular mechanisms that modulate aging and stress responses. Its short lifespan, fully mapped genome and its application in genetic manipulations have enabled researchers to study the function, regulation and output of insulin/IGF-1 signaling (10). This pathway is highly conserved between worms and mammals. *Daf-2* encodes the only insulin/IGF-1 receptor expressed in *C. elegans*. Studies have demonstrated that mutations in *daf-2* increase *C. elegans* resistance to oxidative stress (11) and heat stress (12) and lead to an extended lifespan in a DAF16/FOXO-dependent manner (13). Tullet et al. (14) suggested that the transcription factor SKiNhead (SKN-1/Nrf2) directly integrates insulin/IGF signaling and the stress response. Recent studies have also implicated heat shock factor (HSF) as a regulator of longevity that interacts with the insulin pathway (15,16). MEV-1 is also involved in aging and sensitivity to oxidative stress. The expression patterns of the antioxidant enzymes genes *superoxide dismutase (sod)* and *catalase* (ctl) mirrored one another in the two mutants *daf-16* and *mev-1*. In addition, both strains were extremely sensitive to paraquat, a superoxide anion generator. However, the short life span and oxidative stress-hypersensitivity of *daf-16* mutant may result from suppression of anti-oxidant genes, such as *sod-1* or *sod-3*, rather than increase of ROS production from mitochondria as in *mev-1* (17).

Studies have proposed that integration of cytoprotective and stress-responsive signaling pathways is crucial for environmental adaptation and hence, control of longevity (18). Despite documentation of the many protective properties of the *R. officinalis* extract, there have been no studies on the signaling pathways that may be involved. *C. elegans* has the potential to bridge the gap between *in vitro* and *in vivo* approaches. This model complements genetic studies and helps in the search for a mechanism of action of the extract. In this study, we investigated the effect of eeRo on *C. elegans* stress resistance and longevity and evaluated the signaling pathways involved.

## Material and Methods

### Chemicals and reagents

Ethanol, 5-hydroxy-1,4-naphthoquinone (juglone) and 5-(and-6)-chloromethyl-2′,7′-dichlorodihydrofluorescein diacetate (CM-H_2_DCFDA) were purchased from Sigma-Aldrich (USA).

The eeRo was obtained from the dried leaves (40°C) of this plant, which were collected in the botanical garden of Universidade Federal de Santa Maria, Brazil. The leaves were subjected to an alcoholic extraction (100% ethanol, 1.5 h, 60–70°C) in the Soxhlet apparatus with some modification in relation to the original technique (19). High performance liquid chromatography (HPLC-DAD) was previously performed and revealed the presence of the rosmarinic acid, carnosic acid, chlorogenic acid, caffeic acid, quercetin, rutin and kaempferol (12).

### *C. elegans* strains, maintenance and treatment

The wild-type *C. elegans* strain N2 (Bristol) and mutant worms TK22 [mev-1(kn1)]; CB1370 [daf-2 (e1370) III]; CF1038 [daf-16(mu86)], PS3551 [hsf-1(sy441)]; EU-1 [skn-1(zu67) IV/nT1] and TJ356 [daf-16p::daf-16a/b::GFP + rol-6] were obtained from the *C. elegans* Genetics Center (University of Minnesota, Minneapolis, MN, USA).

For all worms, age-synchronized eggs were obtained by isolating embryos from gravid hermaphrodites using bleaching solution (1% NaOCl, 0.25 M NaOH). For EU-1 worms, we grew synchronized animals and plated young adults. *skn-1* worms are uncoordinated (Unc), while *skn-1* homozygotes are non-Unc, allowing us to enrich for *skn-1* homozygotes using a plate crawling assay. The L1 population was transferred to 10 mL NGM (nematode growth medium) plates seeded with *Escherichia. coli* OP50 as a food source and eeRo at 10, 25 or 50 µg/mL or vehicle (0.1% EtOH) and allowed to develop. The strain CB1370 was maintained at 16°C since it is sensitive to dauer formation at 20°C. The wild-type and the other mutant strains were maintained at 20°C.

### Bacterial growth assay

The minimum inhibitory concentration (MIC) in *E. coli* OP50 was performed according to Clinical and Laboratory Standards Institute with few modifications (20). Bacteria was seeded onto plates with Mueller Hinton agar and allowed to grow for 24 h at 37°C. We then prepared suspensions of microorganisms in Mueller Hinton broth. Fifty microliters of the standardized microorganism suspension was placed in each well of a 96-well microliter plate, along with an equal volume of compound to be tested at different concentrations. We performed broth, growth, and compound vehicle controls to which the results were compared. The plates were incubated for 24 h at 37°C. The MIC was considered as the lowest concentration of the test product able to inhibit the growth of microorganisms evidenced by the use of 2,3,5 triphenyltetrazolium chloride 1%.

### Oxidative stress resistance

Synchronized L1-larva N2 and mutant strains were transferred to treatment plates containing eeRo or vehicle (control) and allowed to develop at 20°C up to adulthood (approximately 2 days). The pretreated worms were collected, washed three times with M9 buffer and transferred into Eppendorf tubes. A volume of 10 µL of 10 mM juglone (stock solution freshly prepared) was added to the micro tubes containing 1000 worms in 990 µL of M9. The worms were exposed to 100 µM juglone (final concentration) during 1 h, washed three times with M9 buffer and transferred to NGM plates containing vehicle or eeRo. After 24 h, the number of living worms was counted and reported as percent of the control. The mutant assays were conducted using the concentration that was deemed most effective in increasing survival (in wild type).

### Thermotolerance assay

Synchronized 1-day-adult N2 and mutant worms, pre-treated with eeRo or vehicle (control) since L1-larval stage, were exposed to 35°C for 4 h. After this procedure the plates were returned to 20°C for 24 additional hours. The number of survivors were scored. The mutant assays were conducted using the concentration that was deemed most effective in increasing survival (in wild type).

### Lifespan assay

The lifespan assay of *C. elegans* was investigated as previously described (21). The pre-fertile period of adulthood was used as time zero (t=0). The worms were kept on NGM plates containing eeRo or vehicle (control) and *E. coli* just in the middle of the plate and transferred to new plates every two days. Nematodes were regarded as dead if they did not move after repeated stimulus. They were excluded if they crawled away from the plate. The maximum lifespan was defined as the 10% of last survival population. The mutant assays were conducted using the concentration that was deemed most effective in inducing lifespan extension (in wild type). Blinding of studies was not possible due to the color of eeRo, which stains the NGM agar. Experiments were performed at least in triplicate with 100 nematodes each.

### Quantification of ROS

Intra-worm ROS generation was measured in *C. elegans* wild-type and mutant strains using CM-H_2_DCFDA, following a previously described method (22) with minor modifications. Briefly, L1 age-synchronized worms were transferred onto culture plates containing either vehicle or eeRo. The worms were maintained at 20°C until adulthood (∼48 h). After, they were collected, washed with fresh M9 buffer three times and transferred to micro tubes. The worms were exposed to 25 mM H_2_O_2_ (final concentration, induced groups) or vehicle (basal groups) during 1 h and washed again with M9 buffer. After, 10 µL of 2 mM CM-H_2_DCFDA were added to the Eppendorf tubes containing 1000 worms in 990 µL M9 (20 μM CM-H_2_DCFDA final concentration) and incubated for 2 h. The mutant worms *mev-1* were just exposed to CM-H_2_DCFDA. The worms were washed and transferred to 96-well plates (100 worms per well). The fluorescence intensity was measured with a plate reader (Excitation: 488 nm; Emission: 510 nm). The mutant assays were conducted using the concentration that was deemed most effective in decreasing ROS levels (in wild type).

### DAF-16 localization

For each slide, a minimum of 20 worms were mounted on 4% agarose pads in M9 and anaesthetized with 10 mM sodium azide. Fluorescence was acquired with an epifluorescence microscope housed in air-conditioned rooms (20°C).

### Statistical analyses

Statistical analyses were performed using GraphPad (Prism for Windows, Version 5.01 GraphPad Software, USA). Significance was assessed by one-way analysis of variance (ANOVA), followed by Newman-Keuls test, or two-way ANOVA, followed by Bonferroni's test for *post hoc* comparison. Significance for survival analysis was assessed by the Kaplan-Meier curve followed by the log-rank test for trend. Values of P<0.05 were considered to be statistically significant.

## Results

### Effect of eeRo on resistance to oxidative stress

**Figure 1:**
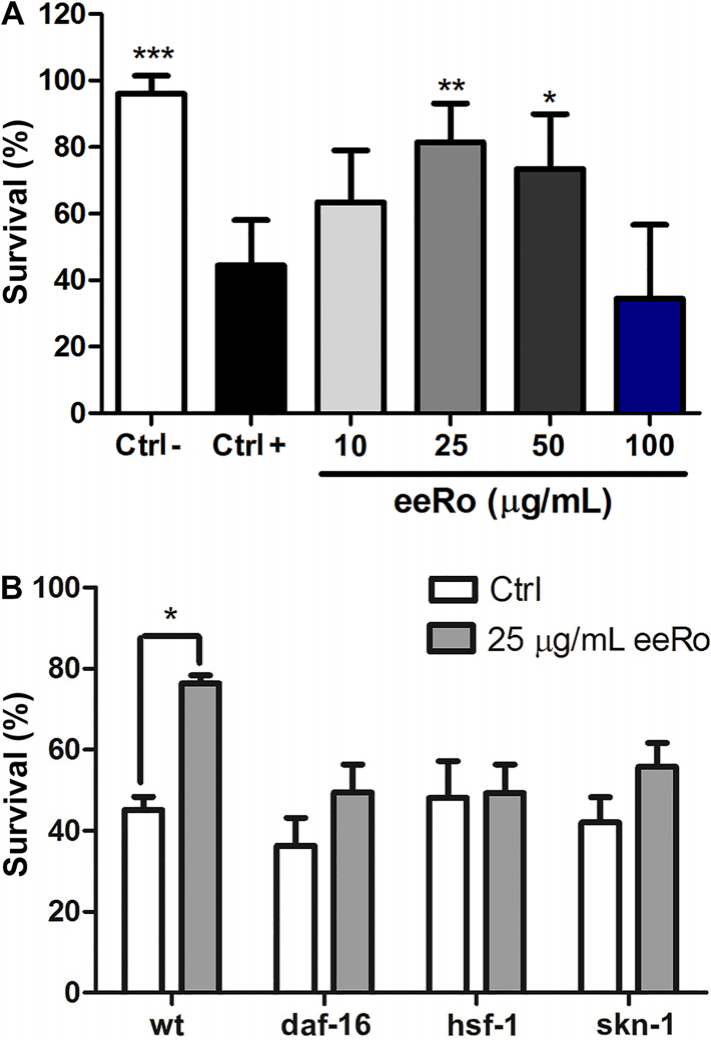
Effect of ethanolic extract of *Rosmarinus officinalis L.* (eeRo) on juglone-induced mortality. Survival of wild-type (*A*) and mutant worms (*B*) treated with eeRo and exposed to 100 µM juglone for 1 h (Ctrl+). Data are reported as percentage of living worms of 100 worms per group in each experiment from 4 independent assays. *A*, *P<0.05, **P<0.01, ***P<0.001, compared to the Ctrl+ group (one-way ANOVA). *B*, *P<0.05 (two-way ANOVA).

To test the antioxidant effect of eeRo *in vivo*, we monitored *C. elegans* survival under oxidative stress. Exposure to 100 µM juglone for 1 h induced an approximately 50% mortality rate in wild-type worms (Figure 1A). Treatment with 10 and 100 µg/mL eeRo did not have any effect on survival rate. In contrast, worms treated with 25 and 50 µg/mL eeRo had a significantly lower mortality rate compared to the untreated induced control group (Figure 1A, Ctrl+). The most effective eeRo concentration in decreasing mortality was 25 µg/mL (P<0.01). We investigated the effect of this dose on the survival rate against juglone exposure in *daf-16, hsf-1* and *skn-1* mutants. We found no significant differences in mortality among the strains evaluated. Furthermore, treatment with 25 µg/mL eeRo did not decrease mortality rate in the mutants, as observed in wild-type worms (Figure 1B).

### Effect of eeRo on thermal tolerance

**Figure 2:**
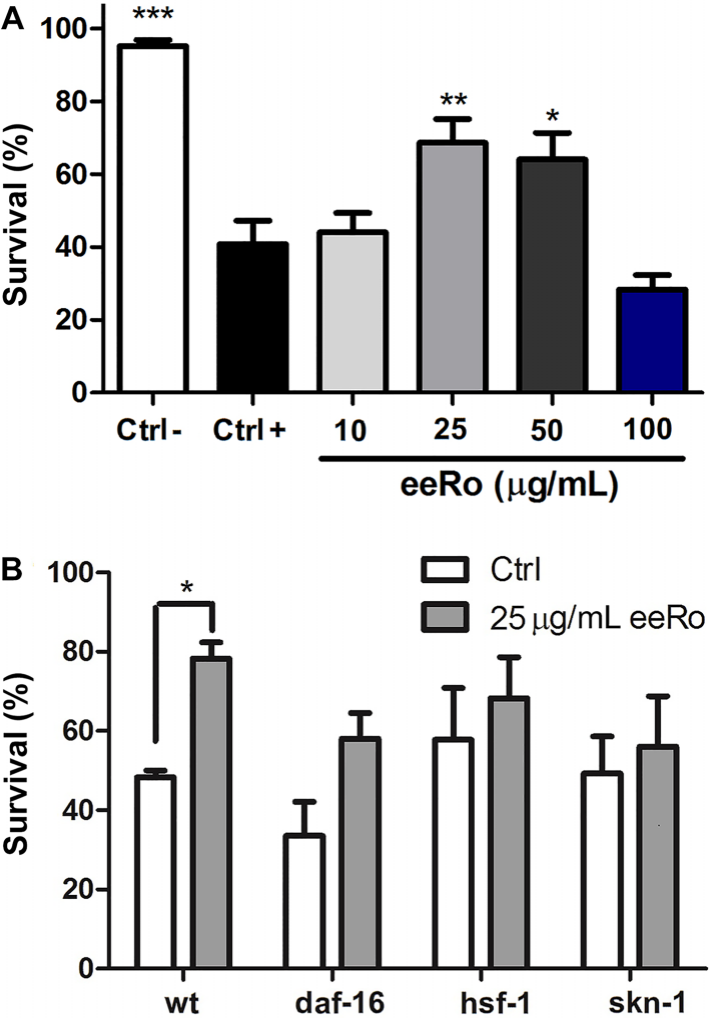
Effect of ethanolic extract of *Rosmarinus officinalis L.* (eeRo) on thermal stress. Survival of wild-type (*A*) and mutant worms (*B*) treated with eeRo and exposed to thermal stress (35°C) for 4 h (Ctrl+). Data are reported as percentage of living worms of 100 worms in each experiment from 4 independent assays. *A*, *P<0.05, **P<0.01, ***P<0.001, compared to the Ctrl+ group (one-way ANOVA). *B*, *P< 0.05 (two-way ANOVA).

Worms were subjected to thermal stress at 35°C for 4 h, which induced a mortality of approximately 50% in wild-type worms (Figure 2A). Treatment with 25 and 50 µg/mL eeRo significantly decreased mortality in 28 and 24%, respectively, in wild-type worms compared to the control group. The most effective concentration of eeRo was 25 µg/mL (P<0.01). We performed the thermal tolerance assay with *daf-16, hsf-1* and *skn-1* mutants. No significant differences in thermal resistance were observed among the untreated strains, although increased mortality was observed in *daf-16*. No significant differences in mortality were noted in *daf-16*, *hsf-1* and *skn-1* mutants treated with 25 µg/mL eeRo compared to the untreated group (Figure 2B).

### Effect of eeRo on ROS production

**Figure 3:**
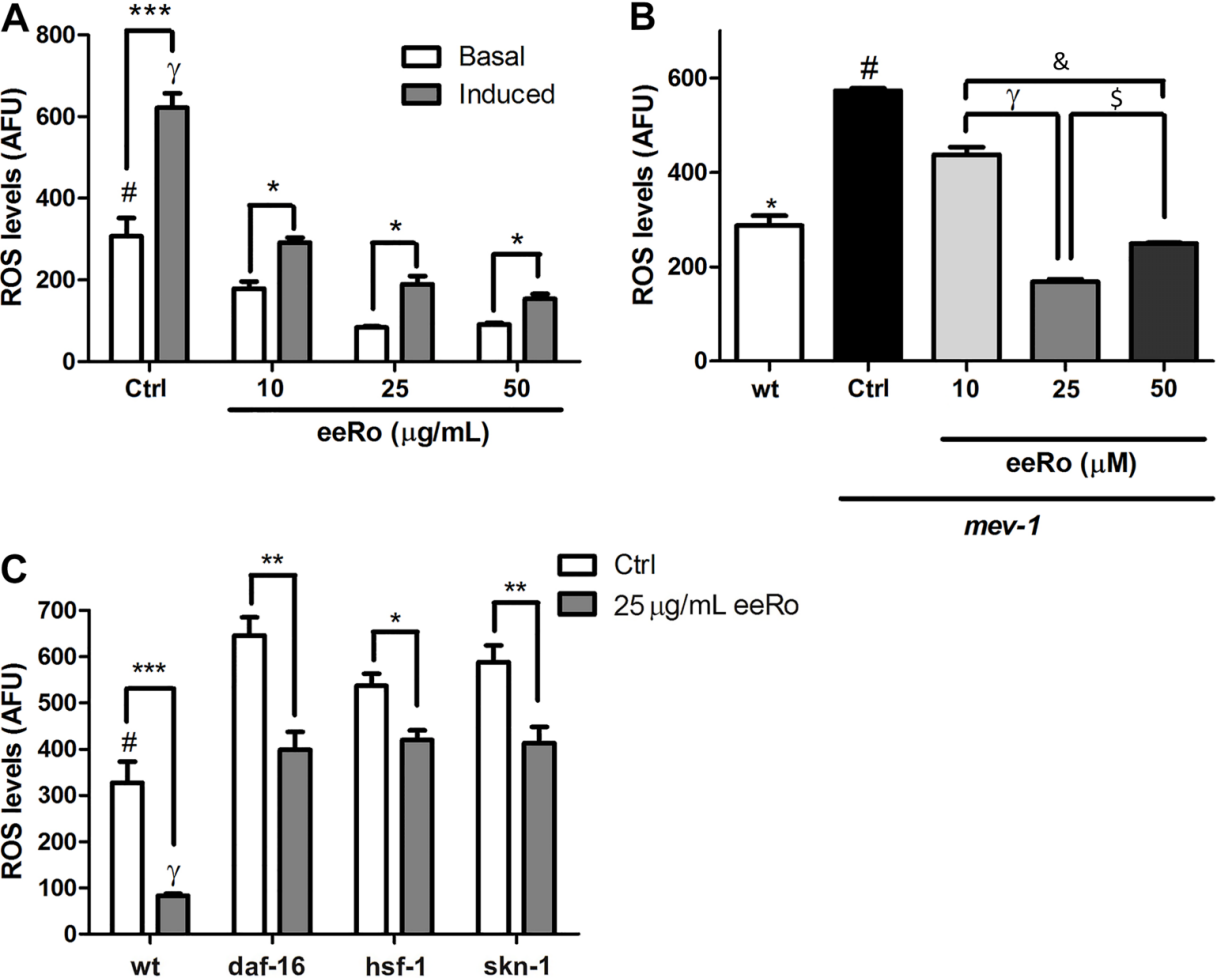
Effect of ethanolic extract of *Rosmarinus officinalis L.* (eeRo) on reactive oxygen species (ROS) production. Data are reported in arbitrary fluorescence units (AFU) from 3 independent assays (n=3). *A*, Levels of basal and H_2_O_2_-induced (25 mM/1 h) ROS production in wild-type (N2) worms. *P<0.001 compared to eeRo-treated basal groups; ^#^P<0.001 compared to eeRo-treated induced groups; ^γ^P<0.01 Ctrl basal compared to Ctrl induced groups. *B*, Levels of ROS production in wild-type (N2) and *mev-1* (TK22) worms. *P<0.001, compared to all other groups; ^#^P<0.001, compared to all other groups; ^γ^P<0.001, 10 µM compared to the 25 µM eeRo treated group; ^$^P<0.001, 25 µM compared to the 50 µM eeRo treated group; ^&^P<0.001, 10 µM compared to the 50 µM eeRo treated group (one-way ANOVA). *C*, ROS levels in *daf-16, hsf-1 and skn-1* mutants treated or not with the extract. *P<0.05, **P<0.01, ***P<0.001, treated compared to untreated group; ^#^P<0.01, compared to the other Ctrl groups; ^γ^P<0.01, compared to the other treated groups (two-way ANOVA).

We used the wild-type strain to investigate if eeRo decreased basal ROS production and ROS induced by H_2_O_2_ exposure. There was a significant increase in ROS levels induced by 1 h exposure to 25 mM H_2_O_2_ (Figure 3A)_._ Worms treated with eeRo at 10, 25, and 50 µg/mL had significantly lower basal ROS production than untreated worms. Moreover, eeRo treatment also prevented an increase in ROS levels induced by H_2_O_2_ in the wild-type strain compared to the untreated induced control group (P<0.05).

We quantified ROS levels in TK22 (*mev-1*) mutant. Figure 3B shows significantly higher ROS levels in the transgenic worms compared to the wild-type strain (P<0.001). At all concentrations tested, the worms treated with eeRo had significantly lower ROS levels compared to the untreated worms (P<0.001). This decrease in ROS levels was most evident at an eeRo concentration of 25 µg/mL.

Measurement of ROS production in *daf-16, hsf-1* and *skn-1* mutants demonstrated that they have higher levels of reactive species than the wild type (Figure 3C). Treatment with 25 µg/mL eeRo significantly decreased ROS levels in these mutants compared to untreated worms (P<0.01). However, the effects of eeRo in the mutants were less apparent than in the wild-type strain (P<0.001).

### Effect of eeRo on DAF-16 translocation

No differences in DAF-16 translocation were observed in worms treated with eeRo compared to untreated worms (data not shown).

### Effect of eeRo on lifespan

**Figure 4:**
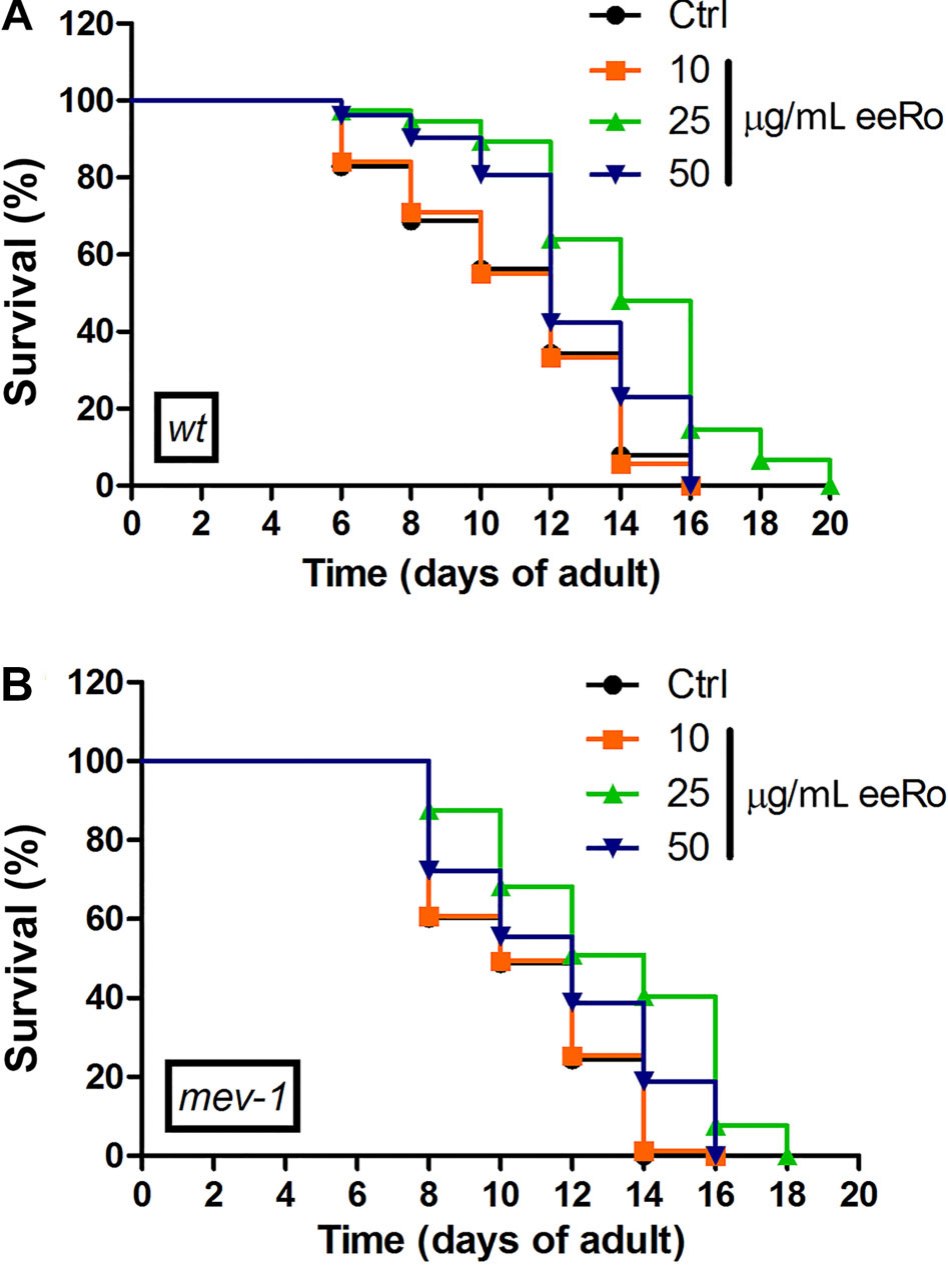
Effect of ethanolic extract of *Rosmarinus officinalis L.* (eeRo) on *C. elegans* lifespan. Survival curves were significantly extended in (*A*) wild-type and (*B*) *mev-1* worms treated with 25 µg/mL eeRo (P<0.0001, log-rank (Mantel-Cox) test).

To test the effects of eeRo on *C. elegans* longevity we monitored time-course survival in wild type and mutant worms. The mean survival time was extended by treatment with 25 µg/mL eeRo in the wild-type strain from 12- (control) to 15-day adults (treated). Similarly, an increase in maximum lifespan also was observed from 16- (control) to 20-day adults (treated) (Table 1). A possible explanation for the beneficial effects of eeRo on aging in *C. elegans* is that these compounds increased cellular stress resistance. To confirm this, we performed a lifespan assay in TK22 (*mev-1*) mutant. *mev-1* had a decreased lifespan compared to the wild type strain. EeRo treatment extended the *mev-1* mean survival-time from 10 to 12 days at a concentration of 25 µg/mL (Table 1). The maximum lifespan of *mev-1* was extended from 16 (control) to 18 days following treatment with 25 µg/mL eeRo (Figure 4B; Table 1).

**Table:**
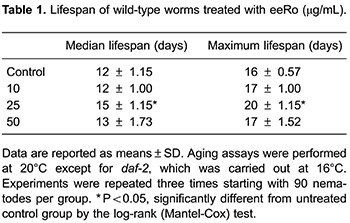

**Figure 5:**
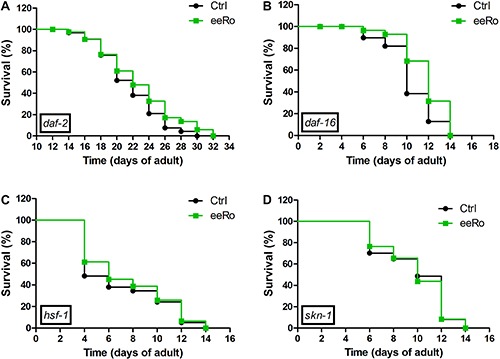
Effect of 25 µg/mL ethanolic extract of *Rosmarinus officinalis L.* (eeRo) in (*A*) *daf-2*, (*B*) *daf-16*, (*C*) *hsf-1* and (*D*) *skn-1* mutants lifespans. Survival curves were significantly different in *daf-16* (median lifespan) and in *daf-2* (maximum lifespan) (P<0.05, log-rank (Mantel-Cox) test).

We performed the lifespan assay in *daf-2*, *daf-16*, *hsf-1* and *skn-1* mutants with and without 25 µg/mL eeRo treatment (Figure 5). EeRo treatment increased the mean lifespan of *daf-16* from 10- (control) to 12-day adults (treated), but not the maximum lifespan. In *daf-2* mutant, we observed a slight increase in the maximum lifespan from 30 to 32 days in worms treated with the extract. EeRo treatment did not have an effect on lifespan in the *hsf-1* and *skn-1* mutants, which had mean survival times of 8 and 9 days, respectively, and maximum lifespans of 13 and 14 days, respectively (Table 2).

**Table:**
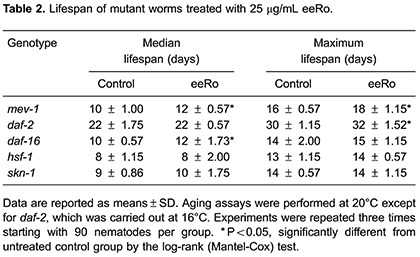

### Effects of eeRo on *E. coli* OP50

To verify whether eeRo influenced *E. coli* OP50 growth, we assessed the minimum inhibitory concentration (MIC). EeRo did not have a significant antimicrobial effect on *E. coli* OP50 growth at a concentration range between 0.39–50 µg/mL (data not shown).

## Discussion

The search for antioxidants from natural sources has received a lot of attention. Antioxidants can inhibit cellular oxidative damage and prevent development of related diseases. The plant *R. officinalis* represents an exceptionally rich source of different bioactive compounds (23). The eeRo used in this study includes flavonoids (quercetin, rutin and kaempferol) and phenolic acids (chlorogenic, caffeic, rosmarinic and carnosic acids) as the most abundant components (9). *R. officinalis* appears to have beneficial effects in prevalent diseases that are strongly linked to aging, such as diabetes and cancer. In the present study, we explored the possibility that this plant can influence aging using the non-parasitic nematode *C. elegans* as a model. We report for the first time that the eeRo increased worm resistance against oxidative and thermal stress and extended *C. elegans* longevity in an insulin/IGF signaling-dependent manner.

Juglone is a naphthoquinone that induces superoxide anion radicals overproduction (24). Juglone can cause premature death at concentrations that overload the organism’s protective capacity (24). As we previously described, 1 h of 100 μM juglone-exposition induces a lethality of approximately 50% of wild-type worms (25). The present study demonstrated that the eeRo treatment was able to decrease the juglone-induced mortality by reducing ROS production and protecting against oxidative damage. These data corroborate the extract antioxidant properties previously mentioned (3). In addition, thermal tolerance was significantly increased by eeRo treatment in wild-type worms (Figure 2A). Previous studies demonstrated that thermal stress causes an increase of ROS levels in the worms and therefore it is likely that the death of worms was at least partially due to oxidative stress (26). These outcomes are known to depend partly on intracellular stress signaling pathways that are activated in response to oxidative stress and as a consequence of direct damage to DNA, proteins, and lipids. These cellular injuries and signaling mechanisms modulate transcription factor activities resulting in changes to gene expression profiles (27). In this way, given that both thermal and chemical stresses result in similar changes in gene expression, eeRo may modulate signaling pathways that are crucial to defense processes. These findings emphasize the potential of eeRo against environmental stress.

Active insulin/IGF signaling promotes phosphorylation-dependent cytoplasmic sequestration of the transcription factors DAF-16/FOXO, HSF-1 and SKN-1/Nrf2 (14,28). Under favorable environmental conditions, signaling through this pathway activates a conserved PI3-kinase/AKT cascade, which causes phosphorylation of DAF-16/FOXO, thereby allowing reproductive development (29). The transcription factor HSF-1 guides DAF-16/FOXO activity and cooperatively induces transcription of a subset of target genes, including heat shock proteins involved in proteostasis (1). SKN-1/Nrf regulates resistance to oxidative stress and expression of detoxification genes (2). We performed survival assays in *daf-16, hsf-1* and *skn-1* mutants and found no significant differences among treated and untreated worms. Moreover, we observed that these strains were more sensitive to oxidative and thermal stress compared to the wild type. These data indicate that DAF-16, HSP-1 and SKN-1 expression, involved in the insulin/IGF signaling network, and the activation of target genes, are essential for eeRo to exert its effect. However, the extract decreased ROS levels in the transgenic strains tested, although less efficiently than in the wild-type. This data suggests that the constituents of the extract may be acting as direct scavengers and then reducing ROS production in the mutants. Besides, more studies are necessary in order to evaluate whether the eeRo capacity to decrease ROS production occurs by modulating the antioxidant system.

In several studies, increased longevity has been closely associated with improved survival under conditions of heat or oxidative stress. In accordance with those studies, eeRo treatment extended mean and maximum lifespan of wild-type worms, likely by increasing cellular stress resistance. Many common stress-induced effects on physiology, gene expression and signaling pathways among animals have been reported (30).

Studies have also demonstrated single gene mutations that influence lifespan. *mev-1* encodes the *C. elegans* ortholog of the succinate dehydrogenase cytochrome *b* subunit, which is required for oxidative phosphorylation. Mutations in *mev-1* result in premature aging and increased sensitivity to oxidative stress (31). Attention has also focused on the insulin-like signaling pathway in *C. elegans* because of its pivotal role in lifespan determination and oxidative stress resistance (32,33). DAF-16 is a well-known regulator of longevity (32). Studies have also implicated heat shock factor (HSF) as an influence on longevity (15). Tullet et al. (14) suggested that SKN-1/Nrf2 directly integrates insulin/IGF signaling and the stress response.

We performed survival assays using *mev-1, daf-2, daf-16*, *hsf-1* and *skn-1* transgenic strains. Activity of the DAF-2 insulin/IGF receptor regulates both L1 arrest and dauer formation in *C. elegans*. Complete loss of *daf-2* function leads to L1 arrest and lethality at 20°C (34). Due to this, we performed the *daf-2* mutant lifespan assay at 16°C. Because the eeRo concentration at 25 µg/mL was most efficient at decreasing the mortality rate and protecting the worms, we performed survival assays with mutants using this dose. We tested *daf-2* mutant in order to investigate if the eeRo effect on longevity was just through this pathway. In *daf-2* and *daf-16* survival curves, there was a slight difference between treated and untreated worms, in a less pronounced way compared to wild type. These data suggest a partial dependence of DAF-2 and DAF-16. These findings suggest that eeRo and *daf-2* mutants may extend adult lifespan through overlapping mechanisms that are not additive. Furthermore, our data demonstrated that DAF-16, HSF-1 and SKN-1 are required for the extract to exert its protective effect given that the treatment did not significantly decrease juglone- or thermal stress-induced mortality in *daf-16*, *hsf-1* and *skn-1* mutants, as observed in wild-type worms. Furthermore, the treatment did not extend *hsf-1* and *skn-1* lifespan, as observed in the wild type worms, emphasizing the need of these target genes for eeRo protective effect.

DAF-16 is crucial for many important processes, including development, stress resistance, thermal tolerance and metabolism (35–38). Along with *hsf-1*, *daf-16* is part of the heat-shock response in *C. elegans* (28). The HSF plays essential and evolutionarily conserved roles in the activation of heat shock-inducible gene expression. HSFs are recognized as regulators of stress-induced gene expression, besides contributing to more complex organismal physiological processes such as development, growth, aging, immunity, and reproduction. We suggest that the beneficial effects of eeRo on aging in *C. elegans* are based on increased cellular stress resistance in a manner partially dependent on IIS pathway activation of target genes. We demonstrated that eeRo modulated the cellular response to oxidative stress in *mev-1* mutants, decreasing ROS levels and extending the lifespan. Previous studies have shown that treatment with natural compounds, such as *Ginkgo biloba* extract, increased *mev-1* mutant resistance to acute oxidative and thermal stress (39). Mutations in *daf-16* and *mev-1* resulted in similar patterns of hypersensitivity, with several interesting differences. The short lifespan and oxidative stress hypersensitivity of the *daf-16* mutant resulted from suppressed anti-oxidant gene expression rather than an increase in ROS production from the mitochondria, as in *mev-1*. In *mev-1* mutant, DAF-16 is present in the nuclei even under normal conditions (40). This observation leads to the prediction that eeRo affects both nematode ROS production and anti-oxidant gene expression.

This study demonstrates the potential protective effect of eeRo in *C. elegans,* as evidenced by an increase in tolerance against oxidative and thermal stress, a decrease in ROS production and extension of longevity in HSF-1- and SKN-1-dependent interactions. These findings suggest that eeRo triggers the signaling pathways that lead to transcriptional activation of downstream targets, which are essential for the effects described in *C. elegans*. These transcription factors play key roles in insulin/IGF-1 signaling, and several additional pathway components have been shown to modulate aging in flies, mice and possibly humans, implying that the effects of the pathway on aging and stress resistance are conserved.

We report for the first time that eeRo increased the resistance against oxidative and thermal stress and extended *C. elegans* longevity in a DAF-16, HSF-1 and SKN-1-dependent manner. These survival-enhancing effects of eeRo on *C. elegans* at both normal conditions and under stress emphasize the potential of *R. officinalis* to promote resistance against oxidative damage in these worms through the activation of related genes. As the death rate of a population is closely related to external stresses, it could be concluded that the survival-enhancing effects of eeRo on *C. elegans* under stress are very important for antiaging research.
